# The Efficacy of Positive Education Intervention for Academic Boredom and Intrinsic Motivation among College Students: A Quasi-Experimental Study

**DOI:** 10.3390/ijerph192013323

**Published:** 2022-10-15

**Authors:** Zheng Jie, Samsilah Roslan, Mohd Mokhtar Muhamad, Mas Nida Md Khambari, Zeinab Zaremohzzabieh

**Affiliations:** 1 Department of Foundation Studies, Faculty of Educational Studies, Universiti Putra Malaysia, Serdang 43400, Malaysia; 2 Institute for Social Science Studies, Universiti Putra Malaysia, Serdang 43400, Malaysia

**Keywords:** academic boredom, class-related boredom, intrinsic motivation, learning-related boredom, positive education

## Abstract

(1) Background: The influence of academic boredom and intrinsic motivation on students’ learning and achievements is receiving more attention from scholars. Nevertheless, studies on how intervention decreases academic boredom and promotes intrinsic motivation during study remain unexplored. (2) Purpose: The purpose of this study is to investigate whether positive education intervention based on the PERMA model would help Chinese college students with learning-related academic boredom, class-related academic boredom, and intrinsic motivation. (3) Methods: This study is quasi-experimental research with a control group including pre-test and post-test. The study was conducted with 173 students, including 86 (n1 = 86) experimental and 87 (n2 = 87) control group students. (4) Results: Results revealed that students in the intervention condition reported significant reductions in learning-related academic boredom and class-related academic boredom, and significant increases in intrinsic motivation in comparison to their counterparts in the control group. (5) Conclusions: These findings indicate that positive education intervention for college students is a promising approach to reducing academic boredom and increasing intrinsic motivation among Chinese college students.

## 1. Introduction

While educational psychologists have long been interested in the research of academic motivation, it is only recently that they have been interested in the study of academic emotions [1]. The cognitive-motivational paradigm focuses on academic emotions that have a favorable or negative influence on academic results, such as achievement, motivation, and deep-level cognitive learning techniques [2]. Academic boredom (AB) as an aversive emotion has been identified as a severe motivating obstacle in higher education (HE) and interferes with students’ learning [3,4]. According to prior studies, the feeling of boredom by students is common at universities and has been associated with several problems that hinder learning [5,6]. AB can be the result of conventional teaching methods. Conventional teaching, or the so-called teacher-centered approach, functions optimally when the teacher can make the lesson engaging. In the absence of this, students could get bored, their thoughts might wander, and they might eventually lose motivation [7]. Thus, the absence of intrinsic motivation (IM) is likely to be reflected in college students’ neglect of their studies [8]. Previous research has found that college students’ academic motivation tends to decline [9]. IM is also linked to academic characteristics, including feeling competent and content with one’s academic experiences, according to Trevino and DeFreitas [10].

University students not only face boredom and a lack of motivation, but they also employ a variety of coping mechanisms to counteract these negative emotions. According to Eren and Coskun [11], coping skills can be used as therapies to avoid the development of serious mental diseases. According to Belton and Priyadharshini [12], fostering an environment wherein students feel empowered and in charge can effectively reduce AB. As stated by Eren and Coskrun [11], students utilize a variety of boredom-coping tactics throughout lessons depending on the context, and the strategies they use may influence their achievement-related outcomes. Westgate and Wilson [13] also verified that there are a variety of ways to reduce boredom and low motivation, suggesting that people might reduce boredom and motivation by aligning activities with important goals or calibrating the cognitive demands of the activity. Even when choices are available, people may not always be aware of the optimal method for reducing boredom in each situation, and even if they are, they may not be able to use it [13]. Martin [14] argued that teachers should focus on the positive aspects of their students as well as their internal characteristics.

Seligman [15] suggested that combining positive psychology components with educational practices is an effective preventative strategy for enhancing students’ mental health and well-being by focusing on the positive characteristics of pupils. The effects of positive educational interventions on students’ mental health outcomes, life satisfaction, and anxiety reduction have been repeatedly demonstrated [16,17]. According to Waters [18], Seligman’s PERMA model is a good foundation for guiding educational settings in using positive education (PE). PERMA essentially stands for Positive Emotion, Engagement, Relationships, Meaning, and Accomplishments. It can be seen as a catch-all word for methods of promoting the well-being of HE students [19,20]. However, nothing is known about the effectiveness of implementing PE intervention for AB and IM among college students, particularly within the Chinese context. Thus, the purpose of this study is to examine the efficacy of PE intervention among Chinese college students to prevent AB and increase IM.

## 2. Literature Review

### 2.1. Boredom in an Academic Setting

AB has been defined as a student’s emotional experience throughout the learning process that is constant but low in relative intensity [21]. It is seen as a negative emotion that diverts one’s focus away from the current work [22]. In comparison to other examined emotions, such as anxiety, Pekrun [21] described boredom as a “silent emotion” (p. 531) as compared to other studied emotions such as anxiety. Boredom is one of the nine academic emotions identified by Pekrun [21] as being widespread among university students. There are two types of AB: class-related boredom (CRB) and learning-related boredom (LRB) [5,21,22]. The term “CRB” refers to a state of boredom that students experience in class because of teaching styles or the environment. LRB is more about the content and method of learning. AB has, therefore, been linked to student retention and learning burnout as well as academic dropout [23]. According to Pekrun [21], 42.2 percent of students reported boredom throughout class-related learning, which is greater than the percentage of undergraduates experiencing anxiety (28.0 percent), anger (19.3 percent), and hopelessness (13.6 percent). Furthermore, in prior studies conducted in China, about half of university students expressed boredom in the classroom at some point [4]. An earlier meta-analysis of 29 studies shows the strength of the negative association between boredom and students’ academic outcomes [5].

### 2.2. Intrinsic Motivation

Intrinsic motivation, often called IM, impacts a person’s attitude toward academic activities and the amount of time and energy they are willing to dedicate to a certain activity [24]. This is also likely responsible for the fact that human learning predominates throughout life [25]. It is a key component of the self-determination theory (SDT) [26] which may roughly be characterized as motivation that arises from the action itself and originates from inside the individual [27]. When students “seek enjoyment, interest, fulfillment of curiosity, self-expression, or personal challenge in the job”, they are intrinsically driven [28]. The benefits of IM are obvious in formal education. In a meta-analysis, Taylor [28] discovered that IM had a significant impact on school achievements. Taylor [29] elaborated on this meta-analysis with studies on university students in Canada and Sweden, showing that IM was consistently connected to better performance, even when baseline achievement was considered.

IM predicts student engagement, which in turn predicts higher achievement, according to Froiland and Worrell [30]. Despite such evidence of IM’s importance, research from a variety of countries suggests that it declines during college years—at least for university-related activities.

### 2.3. Positive Education Intervention

PE incorporates the science of positive psychology in educational settings to improve students’ well-being [14]. It may be seen as a catchall word for methods of promoting students’ well-being in educational contexts. Additionally, PE interventions were utilized to validate positive interventions to improve both academic performance and well-being. The PE movement takes this approach by using positive psychology principles and interventions in the curriculum to promote students’ well-being and academic performance [15], particularly by nurturing the strengths in their characters. For example, Seligman [31] proposed and tested five different positive intervention exercises for one week. The interventions comprised a gratitude visit (focusing on cultivating gratitude), three good things to journal (focusing on increasing positive emotions), you at your best (focusing on identifying character strengths based on personal experience), using signature strengths in a new manner (focusing on identifying and practicing one of the top five-character strengths in a new manner), and identifying signature strengths (focusing on identifying their five highest strengths). Their findings indicate that all five intervention exercises were effective in terms of happiness and depressive symptoms.

**Figure 1:**
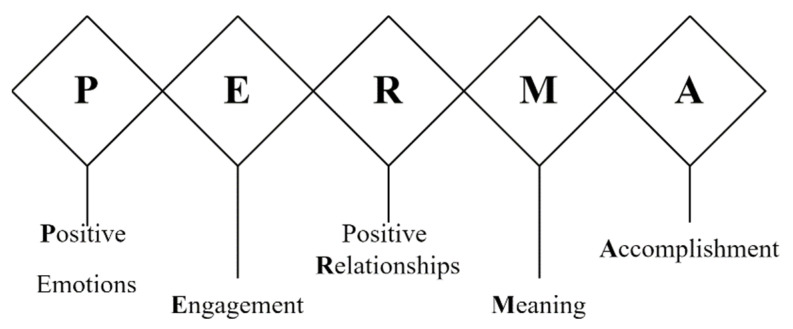
PERMA model [35].

Adler [32] conducted a PE program that aims to teach students ten non-academic life skills, such as empathy and mindfulness, at a large scale in Bhutan, Mexico, and Peru. Their findings demonstrate that students who received PE courses significantly increase their well-being and standardized national exams compared to those in the control group. Seligman [33] investigates interventions that create enabling conditions for life, rather than just interventions that alleviate misery. The use of the PERMA Model to code intervention results could aid in the understanding of AB and IM, as they relate to positive psychology. Seligman conceptualized happiness and well-being as five measurable components of the PERMA model, which is grounded in and based on a positive psychology framework. The PERMA comprises positive emotions, positive engagement, positive relationships, positive meaning, and positive accomplishment. In his description and analysis of a pleasant life, engagement life, and meaningful life, Seligman [34] investigated the effects of optimism and achieving ‘flow’. All three are believed to be crucial for achieving ‘flow’ along with a level of socialness that can be equated to greater happiness. Positive meaning and accomplishment both engage students in activities that benefit a greater cause through their values, while also increasing their confidence and competence through the pursuit and achievement of meaningful outcomes and happiness (Figure 1).

### 2.4. Present Study

Within the Chinese educational setting, the current study aims to include PE interventions in university life. According to the literature, no prior study has been conducted in Chinese universities to implement an intervention to promote IM and minimize AB among Chinese college students. Many studies have stated that interventional research is essential for comprehending the complicated link between PE and students’ well-being. For example, Seligman [15] discovered that numerous interventional programs, such as the Penn Resiliency Program (PRP), have improved students’ overall well-being. Furthermore, Romo-González [35] demonstrated that Psycho-Educative Intervention (PEI) has a positive impact on the happiness and well-being of Mexican university students. As far as the researcher is aware, this is the first study to study the efficacy of PE intervention to increase IM and decrease AB among Chinese college students. This study’s particular aim is to investigate the manner in which PE intervention might help college students reduce AB and boost IM. This is among the early studies that adopted the PERMA model as a PE intervention to increase IM and reduce AB among college students within Chinese contexts. As a result, to fulfill the aforementioned aim, the following research questions were raised:

- Does the effectiveness of a PE intervention lead to an improvement in IM among Chinese university students?
- Does the effectiveness of PE intervention reduce LRB and CRB among Chinese university students?

## 3. Materials and Methods

### 3.1. Study Design

This study aims to evaluate the influence of using positive psychology interventions as part of teaching activities, as compared to teaching as usual, on students’ LRB, CRB, and IM. In this quasi-experimental study, a non-equivalent pre-test and post-test (repeated measurements) control group design was applied [36].

### 3.2. Participants

Students were placed in their current classes by the college administration, which is the reason this particular design was chosen. Due to the use of four intact classes, a true experimental design could not be used. On the one hand, four intact classes were chosen out of ten classes using the fishbowl technique, one of the most popular techniques for drawing a random cluster sample, to reduce the selection bias generated by using non-random intact classes [37]. However, to ensure homogeneity, the pre-test was carried out before the intervention. Selection bias was further minimized by the pre-tests, which showed that the two groups shared similar levels of intrinsic motivation and academic boredom.

**Figure 2:**
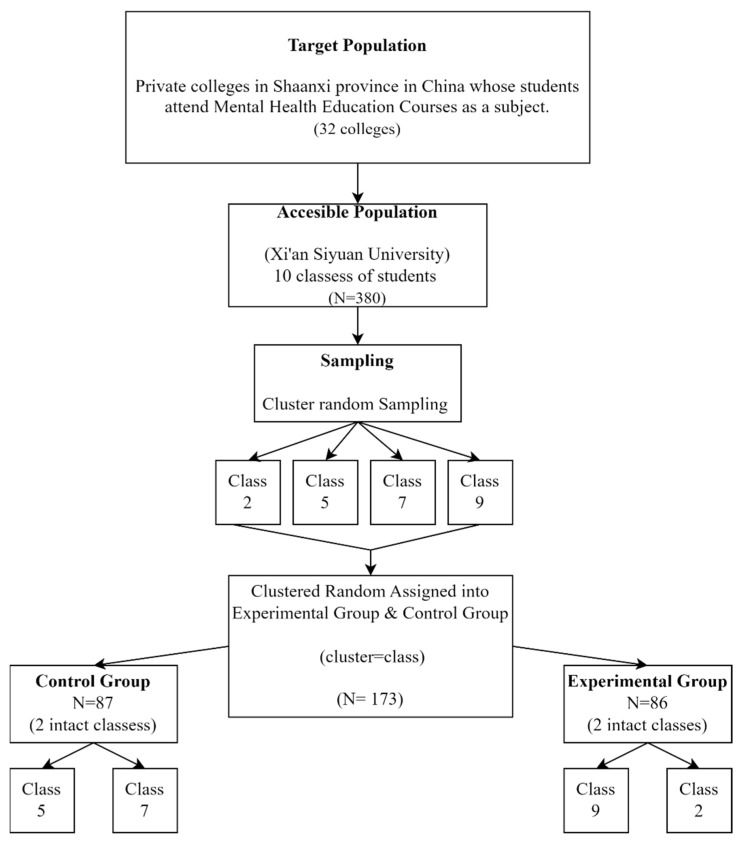
Flow diagram of the recruitment process.

The inclusion criteria were freshmen students who enrolled in the Mental Health Education course in the first semester of the 2021 academic year. Using the statistical program G*Power 3.1.9.7 (Heinrich-Heine-Universität, Düsseldorf, Germany), a target sample size was computed. The significance level was set at 0.05 and the power was set at 80 percent. Figure 2 presents the flow diagram for the recruitment process. In total, 87 students in their second semester served as the control group, while 86 different students in their second semester served as the experimental group. The intervention and data collection occurred in the assigned classroom of the college and took place over 13 weeks, from September 2021 to December 2021 (Table 1).

An independent sample t-test revealed that there were no apparent changes between the experimental and control groups in terms of LRB, CRB, or IM. As shown in Table 1, the score for LRB for experimental group was M = 31.12, SD = 9.08 and for the control group M = 30.76, SD = 8.71; t = 0.24, *p* = 0.79. For CRB, for experimental group was M = 29.98, SD = 9.20 and for the control group M = 29.74, SD = 8.85; t = 0.18, *p* = 0.86. For IM, for experimental group was M = 53.58, SD = 13.80 and for the control group M = 54.76, SD = 13.04; t = −0.58, *p* = 0.57. Thus, the experimental and control groups were homogenous (Table 2).

### 3.3. Intervention

The intervention for this study was a 13-week positive psychology intervention that offered meditation and components of the PERMA model (i.e., Positive emotions, engagement, relationships, meaning, and accomplishment) aimed at reducing AB and fostering IM amongst the students in the experimental group. In this study, a researcher organized each session into four parts: the stages of practicing meditation, constructive thought, skill development, and practice improvement. The researcher began each session with a 5-min meditation practice before the formal class. Interesting videos corresponding to a specific topic were subsequently used to trigger students’ positive emotions. At the next stage, the researcher adopted validated hands-on group activities that made them form embodied memories or practical skills that made them feel useful to further increase students’ motivation and reduce students’ boredom, rather than focusing on correcting their negative views and behaviors.

The researcher expected students to be willing to engage in it. In the last stage, the researcher designed targeted after-class activities to reinforce the effects. Two after-school activities took place in each session, and the students were required to complete one of them to offer them greater autonomy, spark intrinsic motivation, and promote positive action. By doing so, students can not only feel good in class but also act well after class. A brief description of each activity is presented hereafter:

1. Positive emotions: taught students how to foster positive emotions and handle negative emotions using “three good things” and watching the video “amygdala hijack” can help students understand the relationship between negative emotions and the brain. Moreover, the “three-step method (recognition, acceptance, and expression) of coping with negative emotions” can help students take more positive actions.
2. Positive engagement: employed the “Juggling balls in two hands” to guide students through the flow state and outline the criteria required to achieve it. We used the “Microflow” activity to guide students to actively redesign boring situations in learning and daily life to get more flow experience.
3. Positive relationships: taught students how to cultivate and improve interpersonal relationships using an active constructive responding style and empathy, and to actively feel more positive emotions.
4. Positive meaning: guided students to understand and explore their values and goals in life that are more in line with their emotional needs. Employed the “Gratitude visit” activity to arouse students’ good emotional memory so they can start thinking about their purpose and meaning in life. Another group exercise is “Writing your eulogy,” whereby students were encouraged to write their eulogy from the perspective of an outsider to help them identify their life’s meaning.
5. Positive accomplishment: taught students how to strengthen willpower using two group activities. One is “start from five minutes”, in which students were asked to list things that they had to do, but they did not want to, and then pick one of these using “when…, I’ll do…” to imagine when and where they will do it. This would help them save willpower, which leads to positive actions. Another activity is to guide students to learn and use a growth mindset to achieve a sense of fulfillment through practicing a new tongue twister in class.

### 3.4. Dependent Variables and Instruments

#### 3.4.1. Class-Related Academic Boredom

The Achievement Emotions Questionnaire (AEQ) [38] has an eleven-item class-related boredom subscale that was utilized to measure students’ CRB in this study. “The lecture bores me,” and “I consider what else I might be doing instead of sitting in this boring class,” are two examples of the questions. On a five-point Likert scale, responses ranged from one (strongly disagree) to five (strongly agree). A higher overall score indicated a higher level of boredom in the class. In the present study, Cronbach’s alpha was 0.90.

#### 3.4.2. Learning-Related Academic Boredom

The eleven-item LRB scale (AEQ) [2,38] was employed to measure students’ levels of boredom during studying (e.g., “Studying for my courses bores me”), and participants responded on a five-point scale (one being strongly disagreed to five being strongly agreed). Developed under the control-value framework [2,38], the LRB scale is comprised of four components: affective, cognitive, motivational, and physiological. In the current study, internal consistency for baseline scores on the scale was α = 0.80.

#### 3.4.3. Intrinsic Motivation

IM was measured by using a reliable and validated seven-point Likert scale ranging from one (does not correspond at all) to seven (corresponds exactly) with twelve items from Vallerand’s [39] Academic Motivation Scale. The researcher used a Chinese version developed in past studies [40], which displayed acceptable reliability and validity. In this study, Cronbach’s alpha value for the twelve-items IM scale was 0.92.

### 3.5. Procedures

The project received permission from Universiti Putra Malaysia’s Human Research Ethics Committee [Ref No.: JKEUPE-2021-060]. Recruitment of participants and obtaining informed consent were completed. Students were advised that participation in data collection was entirely voluntary and that they would need to provide their consent if they desired to do so. In the first session, the students completed the pre-test survey. Participants in the experimental group began 13 weeks of positive psychology intervention from the second session. Each week’s session lasted 90 min. The researcher led all of the sessions. The students in the control group went to their regular Mental Health Education class. This course was also presented by the researcher utilizing a teacher-centered strategy, which is defined as a traditional educational approach in this study to improve students’ mental health. In session 13, both the control and experiment groups completed the post-test survey after the 13 sessions.

## 4. Data Analysis

To show the difference between the CRB, LRB, and IM scores of the experimental and control groups, a one-way MANOVA was used to assess the effectiveness of the intervention in improving the IM and reducing AB scores of college students. The one-way MANOVA was used to see whether there were any changes in more than one continuous dependent variable between independent groups [41]. 

## 5. Results

Table 2 shows the intercorrelations between all study variables for descriptive purposes. According to Meyers [42], a moderate correlation between the dependent variables is required for MANOVAs to generate appropriate results. Pre- and post-results showed moderate and significant associations between the profile of class-related boredom, learning-related boredom, and intrinsic motivation, indicating that these correlations are accurate enough to proceed with one-way MANOVA (Table 3).

A one-way MANOVA was then calculated to test for differences in LRB, CRB, and IM. Based on the analysis of the assumptions, the data met the criteria of multivariate normality. On a linear combining the three dependent variables, a statistically significant difference was detected between the experimental and control groups, F (3,168) = 8.58, *p* = 0.000; Wilks’ Lambda = 0.72; partial eta squared = 0.29. According to Cohen’s [43] standards, the effect size (partial eta squared) was considered large. When the results in Table 4 for the dependent variables were considered separately, all three dependent variables were found to reach statistical significance, using a Bonferroni adjusted alpha level of 0.01, involving LRB, F (1,171) = 56.93, *p* = 0.000, partial eta squared = 0.25; CRB, F (1,171) = 61.81, *p* = 0.000, partial eta squared = 0.27; IM, F (1,171) = 16.63, *p* = 0.000, partial eta squared = 0.09. According to Cohen’s [43] criteria, the effect size (partial eta squared) obtained for LRB and CRB was large, while IM was moderate.

**Figure 3:**
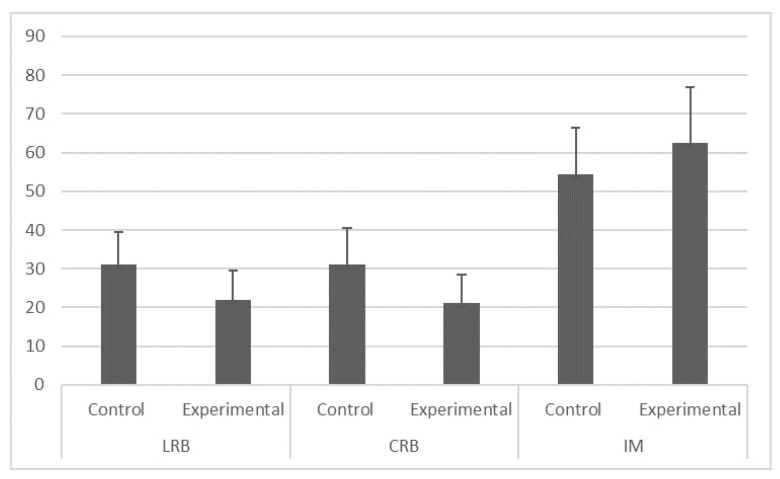
Mean and standard deviation.

Descriptive statistics for LRB, CRB, and IM for the total sample are also presented in Table 4. Table 4 shows that students in the experimental group had significantly lower levels of LRB post-test scores (M = 22.00, SD = 7.59) than students in the control group (M = 31.11, SD = 8.28); significantly lower levels of CRB post-test scores (M = 21.14, SD = 7.24) than students in the control group (M = 31.17, SD = 9.40); and a significantly higher level of IM post-test scores (M = 62.52, SD =14.35) than in the control group (M = 54.30, SD = 12.07) (Figure 3).

## 6. Discussion

The results reported in this current study were that PE intervention based on the PERMA model in the Chinese HE context was effective. This is similar to past results reported in the West for general populations [18,32,44,45]. The reason is that PE moves students in the direction they want to go by providing important components of well-being using positive activities and practical skills, which makes them feel more engaged in the activities and makes the intervention more effective. In this study, the researcher developed a 13-session weekly PE program based on the PERMA model, primarily focusing on using positive activities and practical skills to increase students’ positive emotions and further induce their intrinsic motivation to learn. Each session involves four interconnected stages to make it fun and practical in the classroom teaching and learning process. This would finally trigger an upward spiral of emotional well-being, leading to less academic boredom and improved performance.

Furthermore, the results showed that the intervention program was effective in improving the high levels of AB and low levels of IM of the experimental group. The experimental group showed a significant increase in IM and a decrease in AB, while the control group showed no notable change, indicating that the building of PE intervention contributed significantly to the mental health improvement of those who participated in the program (experimental group). According to the results, IM was significantly predicted by PE. This finding is consistent with prior research that has linked a high degree of PE to higher IM [46,47,48,49].

Other results of this study also showed that PE intervention made a unique contribution to the reduction in LRB and CRB. According to the Control-and-Value theory (CVT) [21], AB is a negative emotion that narrows an individual’s scope of attention, thoughts, and actions, and in turn, affects IM [50,51,52].

According to Fredrickson [53], the PE approach is theoretically effective in reducing academic boredom because positive emotions can reduce negative emotions, and this thus leads to greater performance. In contrast to conventional education approaches, PE approach primarily focuses on using positive activities and practical skills to improve students’ positive emotions or well-being rather than correcting their negative thoughts and behaviors. This broaden and build theory postulates that positive emotions broaden the scope of attention, thoughts, and actions, which helps build enduring personal resources, including psychological resilience, optimism, and creative thinking. In turn, these diverse resources help students better cope with negative emotions and stressful situations in the future, thus leading to greater levels of sustainable well-being [54].

Furthermore, the Three Basic Psychological Needs Theory says that when students have emotions of autonomy, competence, and relatedness, they are intrinsically driven and demonstrate greater well-being [55]. In this study, the researcher designed each session based on those three needs. For example, to cultivate positive engagement in the positive cognition stage, the researcher guided students in experiencing flow by watching “The Monkey Business Illusion” and asked students to focus their attention on one specific task within a limited time. By doing so, students had clear feelings about the flow experience that triggered their interests and increased their perception of competence. Second, in the skills training stage, through the challenge group activity of “Juggling Balls in Two Hands”, the researcher guided students to further experience a flow state in the daily scenario and to share personal feelings in a group as well as summarize the specific conditions that have to be met to achieve flow: clear goals, timely feedback, and the match between challenges and skills. By doing so, students interacted with other students and accumulated positive experiences, which made them feel a sense of fulfillment and belonging.

Furthermore, another in-class activity known as “Micro-Flow Activity” guided students to use the above three conditions to produce flow in redesigning boring situations in daily life, to get more valuable positive life experience, which further satisfied their psychological needs of increasing creative thinking skills or competence. Third, in the practice enhancement stage, there were two after-school activities students could choose to complete. One was to select interesting little practical skills such as juggling balls, to keep practicing for one week, and to experience the flow experience. The other was to let students teach others how to juggle balls with two hands and experience a flow state by teaching. By doing so, the researcher created an autonomy-supportive learning environment for students to develop their self-determination or the perception of autonomy, thereby leading to more positive emotions such as joy and interest as well as establishing an upward spiral of emotional well-being.

Thus, these significant results found for LRB, CRB, and IM supported the Broaden-and-Build theory and Three Basic Psychological Needs theory as well as existing research, which pointed to the benefits of PE [15,45,56]. Therefore, the present results indicated that college students’ IM could be enhanced not just by the expectancies of success or the value of engaging in the task [57], but also by the improvement of their positive emotions, the satisfaction of the three basic psychological needs and the active seeking of a meaningful purpose in an academic experience, which is in line with the PERMA PE model [15].

From a practical perspective, the results of this study could be used in developing and revising the college course for students’ mental health in Chinese college students. This study offers teachers of psychology and counselors systematic teaching implementation plans including a theoretical framework, specific positive activities, supplementary learning materials (e.g., videos, audio, exercises), and teaching procedures regarding how to use the PE approach in class. This positive intervention has the potential to reduce stigma, prevent negative emotions, and ease the burden on mental health services, while at the same time improving students’ positive class experience, enhancing IM, and fostering positive emotions. Thus, this is a win-win situation when improving academic performance and well-being skills.

In addition, in contrast to the traditional education approach that often teaches students how to correct their irrational thoughts and behaviors, the PE approach focuses on adopting positive activities and practical skills to satisfy basic psychological needs (autonomy, competence, relatedness), which further enhances students’ IM. Thus, students are more engaged in those positive activities and the whole class becomes incredibly positive and proactive. Thus, a PE approach is an effective way to practice the whole-student approach and improve education for sustainability as it enhances both learning skills and well-being. Finally, introducing this intervention to college students could help them learn PE approaches and teaching methods, which are greatly beneficial to their future careers.

## 7. Limitations and Future Studies

This study has many limitations that must be acknowledged. The current study has various confounding and uncontrolled factors, such as the time of day the intervention was provided. These types of variables are popular in quasi-experimental field investigations. Using a controlled laboratory environment, the intervention is provided to exactly one person at a time, similar to many psychological studies. This is one way to decrease the number of confounding variables. This, however, is less natural than the classroom atmosphere, which is a trade-off and does not address the issues with the time between therapy and follow-up. Furthermore, the intervention can be subject to demand characteristics [58], especially emphasizing positive factors that were included in the self-report measures. Qualitative research analyzing the teaching process and students’ individual experiences while in class using observation, interviews, or experience sampling methods would provide a more comprehensive evaluation of the effects of this PE program on university students. Because the technique was not designed to actively incorporate and examine the effect of the intervention across varied languages, cultures, and socioeconomic groups, the generalizability of these results may be limited.

As part of this, it is critical to collect data on the university’s environmental features as well as other related social and emotional skills based on projects or curricular interventions, how they work within the PERMA framework, and their overall influence on the university’s outcomes. Furthermore, the researcher adopted the PERMA model to design the whole program, which involves many corresponding positive activities and practical skills. Thus, more studies are needed to ascertain whether this unique PE model is also suitable for other cultures distinct from Chinese culture. The researcher encourages the implementation of this PE intervention within HE in other cultures or through different delivery formats such as online teaching, especially during the COVID-19 pandemic to enable a comprehensive understanding of the interaction between culture, delivery formats, and this PE program. Further, this study did not embed the PE intervention into other academic subjects such as physical education, music, and drawing. Thus, future research can investigate the effect of PE intervention embedded in other subjects or classes, which can further reduce stigma and improve obedience to experiments.

## 8. Conclusions

The current study tried to construct a PE intervention for Chinese college students based on the PERMA model as a way to decrease or prevent AB and boost IM. The intervention resulted in significant changes in the AB and IM scores of college students. There were no statistically significant changes in the scores of the control group. This work is expected to contribute to a better understanding of AB and IM among Chinese college students, as well as the intervention used to alleviate and motivate feelings. This work, hopefully, will serve to pave the way for future research into AB and IM among Chinese college students and other populations. Furthermore, it is hoped that this intervention will give academic professionals and education officials significant insight into addressing the problem of boredom and low motivation among Chinese college students.

## Figures and Tables

**Table 1:** Demographic characteristics of participants according to gender, academic background, and age (N = 173).

Variables	Total	Experimental Group	Control Group
	Frequency (%)	Frequency (%)	Frequency (%)
**Gender**			
Female	139 (80.3)	68 (79.1)	71 (81.6)
Male	34 (19.7)	18 (20.9)	16 (18.4)
**Age**			
17 years old	3 (1.7)	2 (2.3)	1 (1.1)
18–20 years old	161 (93)	80 (92.9)	81 (93.1)
21 years and over	9 (5.2)	4 (4.7)	5 (5.7)

**Table 2:** The pre-test of experimental and control groups on LRB, CRB, and IM.

	Group	N	M	SD	SEM	t	*df*	*p*
LRB	Experimental	86	31.12	9.08	0.98	0.24	171	0.79
	Control	87	30.76	8.71	0.93			
CLR	Experimental	86	29.98	9.20	0.99	0.18	171	0.86
	Control	87	29.74	8.85	0.95			
IM	Experimental	86	53.58	13.80	1.49	−0.58	171	0.57
	Control	87	54.76	13.04	1.40			

Note. Learning-related boredom = LRB; Class-related boredom = CRB; Intrinsic motivation = IM.

**Table 3:** Correlation for all variables used in these analyses.

No.	Scale	1	2	3	4	5
1	LRB scale (pre-test)					
2	CRB scale (pre-test)	0.741 **				
3	IM scale (pre-test)	−0.496 **	−0.512 **			
4	LRB scale (post-test)	0.510	−0.625	0.544		
5	CRB scale (post-test)	−0.628	−0.515	0.515	0.831 **	
6	IM scale (post-test)	−0.577	0.637	0.609	−0.518 **	−0.596 **

Note. Class-related boredom = CRB, learning-related boredom = LRB, Intrinsic motivation = IM, ** *p* < 0.01.

**Table 4:** Mean and Standard Deviation (Mean ± SD) and summary of the analysis.

	Control	Experimental	Delta	α		*F*	*p*	η_p_^2^
	Mean ± SD	Mean ± SD	Mean ± SD	Control	Experimental			
LRB	31.11 ± 8.28	22 ± 7.59	−9.11 ± 0.66	0.94	0.91	56.93	0.000	0.25
CRB	31.17 ± 9.40	21.14 ± 7.24	−10.03 ± 2.16	0.81	0.90	61.81	0.000	0.27
IM	54.30 ± 12.07	62.52 ± 14.35	8.22 ± 2.28	0.88	0.91	16.63	0.000	0.09

Note. Learning-related academic boredom = LRB; Class-related academic boredom = CRB; Intrinsic motivation = IM. N = Number of respondents; SD = Standard deviation.

## Data Availability

The raw data supporting the conclusion of this article will be made available by the authors, without undue reservation.
